# The Book World of Medicine and Science

**Published:** 1901-08-03

**Authors:** 


### The Book World of Medicine and Science.

The No-Breakfast Plan and the Fasting Cure. By Edward Hooker Dewey, M.D. (Meadville, Pa., U.S.A.: Published by the Author. London: Gay and Bird. 1900. Price 4s. net.)

This is a little book which first gives a portrait of the author, then his autobiography, and then proceeds to enlighten a more or less sceptical world as to the advantages of going without food. In its production the author has succeeded in writing an enormous amount of unmitigated nonsense. We do not pretend to believe what he tells us about the fasts made by his patients, nor what his photographs tell as to the condition in which these "fasting" people found themselves after enjoying six weeks more or less of complete starvation. People have been taken in by neurotics before, and, to put the most charitable construction on Dr. Dewey's writings, we can but suppose that he also has made mistakes like many a better man. All the same, notwithstanding the quackery with which this book is pervaded, it does give emphasis to two points in treatment which we cannot but think are far too often overlooked or neglected, notwithstanding their obviousness—namely, in the first instance, that a considerable amount of energy is used up in the mere digestion of food, so that the highest effort in other directions cannot be made while digestion is going on; and, secondly, the still more patent fact that when food cannot be digested it is best that it should not be taken. From the first of these comes the "no-breakfast plan."

If the reader has patience, however, to wade through the padding in this book, he will find that even the term "no breakfast" must be taken with a grain of salt; for "one need not always wait until noon to eat the first meal," and one is allowed a cup of coffee before that, so that the "no-breakfast" person is not so badly off after all. The book hails from America, and is probably to be taken as a not entirely unreasonable revolt against the American breakfast. As to the fasting cure, whether one may properly accept Dr. Dewey's teaching depends much upon how far we believe his statements in regard to the prolonged fasts undertaken by his patients, and the amount of work done by them without food; and under these circumstances it is to be regretted that he has chosen to state his facts in such a way as not greatly to encourage acceptance by careful men.

The Practical Guide to the Public Health Acts: A Vade Mecum for Officers of Health and Inspectors of Nuisances. By Thomas Whiteside Hime, B.A., M.D. Second, much enlarged, Edition. (London: Ballière, Tindall & Cox. 1901. Price 15s. net.)

At first sight this book bears the aspect of a mere compendium of laws—a book for reference and reference alone. On diving somewhat further into its pages, however, it is soon found to be anything but dull reading, the comments on the Acts referred to being full of acute criticism; and Part III., which deals with "Miscellaneous Memoranda," is crammed with information on matters interesting to health officers. In Part I., which occupies about 200 pages, we find the text of such portions of the following Acts as especially concern medical officers of health and inspectors of nuisances—namely, the Public Health Act, 1875; Infectious Disease (Notification) Act, 1889; Infectious Disease (Prevention) Act, 1890; and Public Health Acts Amendment Act, 1890. All these Acts are fully annotated, cases, decisions, explanations, and cross-references being given in abundance. In Part II. a large number of Acts of Parliament are dealt with in a shorter manner, only certain portions being given in full. Much judgment has been exercised in the selection of the parts of which the full text is given, and to the medical officer who has not access to a library containing the Acts themselves, this part of the book is likely to prove of great value from the large number of statutes referred to, many of which, although bearing upon public health, could hardly be called Public Health Acts. Part III. is founded upon various memoranda issued from time to time by the Local Government Board, but so completely are these subjects considered that this portion of the book really takes the form of a manual of public health administration. Following this is an appendix of 223 pages, containing legal cases and a variety of other matters for which place has not been found elsewhere.

No doubt there are points in this book with which it would be easy to find fault, more especially in regard to the occasional intrusion of the author's opinion in places where one might look rather for a colourless *résumé* of the law. Still all this sort of thing does at least give life to the book. One feels as one reads its pages that it is not a mere compilation made to order, but is the outcome of a large experience in the practical working of sanitary administration; and although, perhaps more than some authors, Dr. Whiteside Hime shows the absurdities which have been embodied in certain Acts of Parliament, and although much more than most sanitary officials he sees the weak spots which too often exist in the relation between medical officers of health and the practitioners in their districts, we cannot say that he is wrong in these matters, or that in his criticism he goes beyond what is the truth. Take it altogether we think that Dr. Whiteside Hime has written a book which admirably serves its purpose: its annotations are to the point, its cross references are copious, and the general information is certainly such as would not easily be obtained in any other work of the same size on the subject.

